# Active Biomedical Materials and Their Applications

**DOI:** 10.3390/jfb15090250

**Published:** 2024-08-30

**Authors:** Christie Ying Kei Lung

**Affiliations:** Restorative Dental Sciences, Faculty of Dentistry, The University of Hong Kong, Hong Kong, China; cyklung@hku.hk

Active biomedical materials are designed to heal and restore the functions of people recovering after injuries or diseases. They are used to repair or replace a specific biological function of dysfunctional tissues (Figure 1). They are synthetic or natural materials, such as metals and their alloys, ceramics, composites, or polymers. They must meet several criteria, such as having good mechanical properties, excellent biocompatibility, high corrosion resistance, good tribological properties, and non-toxicity. The modification of existing biomedical materials and the development of new biomedical materials are effective strategies for improving material performance. 

This Special Issue, “Active Biomedical Materials and Their Applications”, provides updated information on the synthesis and properties of different materials, namely ceramics, polymers, nanoparticles, and composites, and their applications. It collects seven novel studies and four review articles. Experts from different research fields share their ideas and report their findings on these topics. 

In the study by Tian, X. et al. (contribution 1), an injectable bioactive and a degradable calcium sulfate/hydroxyapatite (CaS/HA) cement was developed for the augmentation of fenestrated pedicle screws in an osteoporotic spine [2]. The results showed that CaS/HA augmentation was effective in enhancing the pull-out force of the fenestrated pedicle screws in osteoporotic vertebrae in comparison with non-augmented screws. This technique allowed for adequate cement distribution and the interdigitation ability of the CaS/HA cement in the trabecular bone. 

In the review by Liu, D. et al. (contribution 2), an overview of currently used biodegradable bone cements, such as calcium phosphates, calcium sulfates, and organic–inorganic composites, was described [3]. The degradation mechanism and the clinical performance of the biodegradable cements were discussed in detail.

In the review by Itzhaki, E. et al. (contribution 3), the synthesis and characterization of proteinoid polymers and the nanocapsules containing synergistic drugs, cannabinoids, and TRAIL for cancer theranostics were summarized [4]. Proteinoids are random polymers composed of amino acids synthesized using stepwise thermal polymerization. The in vitro and in vivo studies of the latest findings in this area were discussed. 

In the review by Sousa, A.B. et al. (contribution 4), the authors discussed the potential applications of specialized pro-resolving mediators (SPMs) in the development of new immunomodulatory biomaterials [5]. These mediators are a family of endogenous molecules that include lipoxins, resolvins, protectins, maresins, Cysteinyl-SPMs, and n-3 docosapentaenoic acid-derived SPMs. SPMs have important anti-inflammatory and pro-resolutive actions, such as decreasing the recruitment of polymorphonuclear leukocytes, inducing the recruitment of anti-inflammatory macrophages, and increasing macrophage clearance of apoptotic cells through a process known as efferocytosis. 

In the study by de Araujo, M.M. et al. (contribution 5), the authors develop solid lipid-polymer hybrid nanoparticles (SLPHNs) as topical delivery systems for small interfering RNA (siRNA) molecules [6]. Their findings showed that the developed SLPHN–0.25% polyethyleneimine was a promising nanoplatform for the cutaneous delivery of siRNA, as it favored the ability of siRNA to penetrate the skin through the stratum corneum barrier and kept it in the epidermis.

In the study by Tărăboanță, I. et al. (contribution 6), a light-activated microhybrid composite resin incorporated with green silver nanoparticles was developed to improve the antibacterial activity and surface hardness of the resin composite [7]. The authors reported that the resin composite loaded with silver nanoparticles inhibited the Streptococcus mutans activity, but there was no improvement in surface hardness. 

In the study by Lin, C.J. et al. (contribution 7), an ultrasonic-assisted digestion of a formic acid decellularized extracellular matrix (UdECM) hydrogel was developed for diabetic wound treatment [8]. The wound-healing effect of the UdECM hydrogel incorporated with rat platelet-rich plasma (R-PRP) and sacchachitin nanofibers (SCNFs) on diabetic wounds was examined using an animal model. The results showed that the UdECM/SC/R-PRP group improved diabetic wound healing and the recovery of diabetic wounds in normal tissue. 

In the study by Chauvin, A. et al. (contribution 8), a hydroxyapatite-based coating on zirconia was designed [9]. The zirconia was coated with polyethyleneimine-hydroxyapatite (PEI-HAp) followed by thermal treatment. The results showed that the adhesion of hydroxyapatite on zirconia was improved. 

In the study by Sung, P. et al. (contribution 9), a network structure of anatase- and rutile-type TiO_2_ with CuO fine particles was formed on titanium when it was subjected to H_2_O_2_-Cu(OAc)_2_-heat treatments [10]. The results showed that the coated layer exhibited good antibacterial activity against E. coli and S. aureus when subjected to visible-light irradiation. This was due to the generation of reactive oxygen species and hydroxyl free radicals (·OH) under visible-light irradiation. 

In the study by Soto ER et al. (contribution 10), they developed a new drug delivery system, yeast particles for the encapsulation of fungicides, tetraconazole (TET), and prothioconazole (PRO), with a high payload-loading capacity, encapsulation efficiency, and encapsulation stability [11]. The activity of the yeast particles in encapsulated PRO on azole-resistant C. albicans strains was enhanced compared to unencapsulated PRO. 

In the review by Abdalla, M.M. et al. (contribution 11), the authors provided a comprehensive review of the applications of strontium compounds in dentistry [12]. Their comprehensive summary elucidated the uses and benefits of strontium, the mechanisms of its biological interactions, and its applications in dentistry. 

In summary, the articles included in this Special Issue, “Active Biomedical Materials and Their Applications”, report new data and contribute to recent developments in biomedical materials. Hopefully, these contributions will both practically benefit the readership and encourage further research in the field of biomedical materials.

I extend my gratitude to the authors, the reviewers, and the MDPI *JFB* editors and their team for their invaluable contributions. 

## Figures and Tables

**Figure 1:**
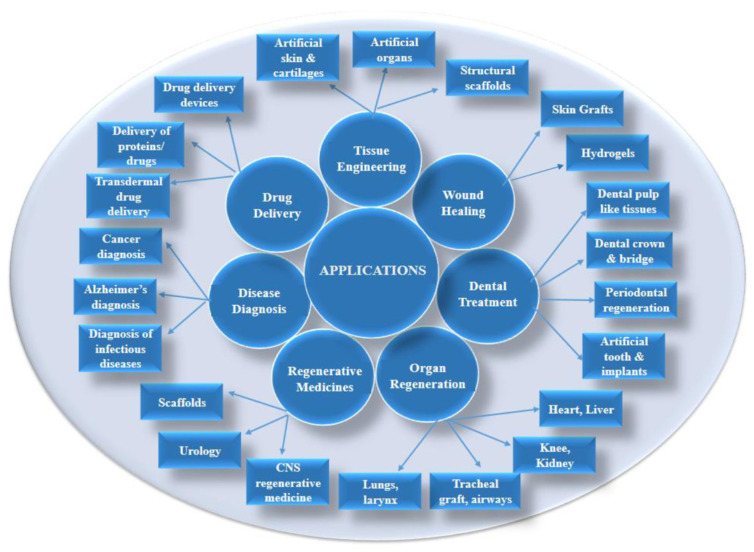
Applications of biomedical materials in different areas. Reprinted from Ref. [1].

## References

[1] Nikita N., Kamlesh W., Milind U. (2021). An Overview on Biomaterials: Pharmaceutical and Biomedical Applications. J. Drug Deliv. Ther..

[2] Tian X., Raina D.B., Vater C., Kilian D., Ahlfeld T., Platzek I., Nimtschke U., Tägil M., Lidgren L., Thomas A. (2022). Evaluation of an Injectable Biphasic Calcium Sulfate/Hydroxyapatite Cement for the Augmentation of Fenestrated Pedicle Screws in Osteoporotic Vertebrae: A Biomechanical Cadaver Study. J. Funct. Biomater..

[3] Liu D., Cui C., Chen W., Shi J., Li B., Chen S. (2023). Biodegradable Cements for Bone Regeneration. J. Funct. Biomater..

[4] Itzhaki E., Elias Y., Moskovits N., Stemmer S.M., Margel S. (2023). Proteinoid Polymers and Nanocapsules for Cancer Diagnostics, Therapy and Theranostics: In Vitro and In Vivo Studies. J. Funct. Biomater..

[5] Sousa A.B., Barbosa J.N. (2023). The Use of Specialized Pro-Resolving Mediators in Biomaterial-Based Immunomodulation. J. Funct. Biomater..

[6] de Araujo M.M., Borgheti-Cardoso L.N., Praça F.G., Marcato P.D., Bentley M.V.L.B. (2023). Solid Lipid–Polymer Hybrid Nanoplatform for Topical Delivery of siRNA: In Vitro Biological Activity and Permeation Studies. J. Funct. Biomater..

[7] Tărăboanță I., Burlec A.F., Stoleriu S., Corciovă A., Fifere A., Batir-Marin D., Hăncianu M., Mircea C., Nica I., Tărăboanță-Gamen A.C. (2023). Influence of the Loading with Newly Green Silver Nanoparticles Synthesized Using Equisetum sylvaticum on the Antibacterial Activity and Surface Hardness of a Composite Resin. J. Funct. Biomater..

[8] Lin C.-J., Lin H.-L., You W.-C., Ho H.-O., Sheu M.-T., Chen L.-C., Cheng W.-J. (2023). Composite Hydrogels of Ultrasound-Assisted-Digested Formic Acid-Decellularized Extracellular Matrix and Sacchachitin Nanofibers Incorporated with Platelet-Rich Plasma for Diabetic Wound Treatment. J. Funct. Biomater..

[9] Chauvin A., Garda M.-R., Snyder N., Cui B., Delpouve N., Tan L. (2024). Hydroxyapatite-Based Coatings on Silicon Wafers and Printed Zirconia. J. Funct. Biomater..

[10] Sung P.-C., Yokoi T., Shimabukuro M., Mokudai T., Kawashita M. (2024). Apatite-Forming Ability and Visible Light-Enhanced Antibacterial Activity of CuO-Supported TiO_2_ Formed on Titanium by Chemical and Thermal Treatments. J. Funct. Biomater..

[11] Soto E.R., Rus F., Ostroff G.R. (2024). Yeast Particle Encapsulation of Azole Fungicides for Enhanced Treatment of Azole-Resistant Candida albicans. J. Funct. Biomater..

[12] Abdalla M.M., Sayed O., Lung C.Y.K., Rajasekar V., Yiu C.K.Y. (2024). Applications of Bioactive Strontium Compounds in Dentistry. J. Funct. Biomater..

